# Sarcopenia and Myosteatosis as a Predictor of Post-Operative Outcomes in Patients Undergoing Laparotomy for Abdominal Emergencies

**DOI:** 10.3390/jcm14186639

**Published:** 2025-09-20

**Authors:** Simone Giudici, Ezio Lanza, Ludovica Lofino, Alberto Barison, Angela Ammirabile, Giulia Mauri, Davide Zulian, Martina Ceolin, Andrea Brocchi, Daniele Del Fabbro

**Affiliations:** 1 Emergency and Trauma Surgery Unit, Department of General Surgery, IRCCS Humanitas Research Hospital, via Manzoni 56, 20089 Rozzano, MI, Italy; simone.giudici@humanitas.it (S.G.); davide.zulian@humanitas.it (D.Z.); martina.ceolin@humanitas.it (M.C.); andrea.brocchi@humanitas.it (A.B.); 2 Department of Diagnostic and Interventional Radiology, IRCCS Humanitas Research Hospital, via Manzoni 56, 20089 Rozzano, MI, Italy; ezio.lanza@hunimed.eu (E.L.); ludovica.lofino@humanitas.it (L.L.); alberto.barison@humanitas.it (A.B.); angela.ammirabile@humanitas.it (A.A.); 3 Department of Biomedical Sciences, Humanitas University, via Rita Levi Montalcini 4, 20072 Pieve Emanuele, MI, Italy; giulia.mauri@humanitas.it

**Keywords:** emergency laparotomy, myosteatosis, sarcopenia, surgical outcomes

## Abstract

**Background**: Emergency laparotomy (EL) is related to a high risk of morbidity and mortality. Sarcopenia (low skeletal muscle mass) and myosteatosis (poor muscle quality) have emerged as prognostic indicators in various clinical contexts. This study evaluated the impact of these conditions on postoperative outcomes in patients undergoing EL for abdominal emergencies. **Methods**: A retrospective analysis was conducted on 242 patients who underwent EL between January 2016 and December 2023. Skeletal muscle index (SMI) and muscle radiation attenuation (MRA) were measured using CT imaging at the L3 level. Sarcopenia was defined as SMI ≤ 41.6 cm^2^/m^2^ for men and ≤ 32 cm^2^/m^2^ for women. Myosteatosis was defined as MRA ≤ 29.3 HU for men and ≤ 22 HU for women. Outcomes included 30-day mortality, hospital length of stay (h-LOS), severe complications (Clavien-Dindo ≥ 3), and Intensive Care Unit (ICU) admission. **Results**: Of the 242 patients (median age: 70; 51.2% men), 42.6% were sarcopenic and 78.1% had myosteatosis. Sarcopenia was not significantly associated with any postoperative outcomes. Conversely, myosteatosis was significantly associated with longer h-LOS (17 vs. 8 days; *p* < 0.001), higher rates of severe complications (37.1% vs. 22.7%; *p* = 0.048), and ICU admission (48.2% vs. 28.3%; *p* = 0.010), but not with 30-day mortality. Multivariate analysis confirmed myosteatosis as an independent predictor of prolonged hospital stay (HR 0.59, 95% CI: 0.42–0.84 *p* = 0.003). **Conclusions**: Myosteatosis, rather than sarcopenia, is associated with worse postoperative outcomes following EL for abdominal emergencies. Including myosteatosis in preoperative risk assessments may improve the identification of high-risk patients and guide perioperative management.

## 1. Introduction

Emergency laparotomy (EL) is associated with some of the highest incidences of morbidity, disability, and mortality among all surgical interventions [1]. Predicting outcomes following EL based on preoperative data remains challenging due to the complexity of patients’ conditions, the variety of surgical techniques employed, and the limited time available for preparation.

Various scoring systems have been utilized in previous studies to predict the risk associated with EL [2,3]. However, many of these systems may have limited utility in emergency settings because most do not incorporate diagnostic findings and partly rely on subjective surgical judgment criteria, which can lead to variability.

Over recent years, body composition has gained increasing importance in predicting surgical treatment outcomes, particularly in relation to sarcopenia and myosteatosis. Sarcopenia, as defined by the European Working Group on Sarcopenia in Older People, is characterized by low muscle mass (below the 5th percentile) combined with reduced muscle function (strength or performance). While typically observed in elderly populations, it is also prevalent among oncologic patients and those with other diseases [4]. Sarcopenia is a known contributor to the development of physical frailty: in fact, primary sarcopenia usually precedes frailty, whereas frailty itself is the risk factor for disability and eventually death. The underlying mechanisms of sarcopenia and, consequently, frailty are mostly catabolic mechanisms and degeneration of organ systems and muscles, which lead to a decreased ability to withstand physical stressors, as a surgical intervention, for instance [5].

Myosteatosis refers to the ectopic deposition of fat within muscle tissue, which increases with aging and is associated with changes in both total and regional fat distribution [6]. Myosteatosis seems to be related to increased frailty and decreased muscle and mobility function in both the upper and lower extremities.

Previous studies have demonstrated that sarcopenia and myosteatosis are linked to a higher risk of postoperative complications and decreased long-term survival after oncologic surgery [7,8]. Similar results could also be expected in emergency surgery: pre-surgical assessment of muscle mass and quality could help identify high-risk patients to enable personalized perioperative management strategies. However, their role in this setting remains less well defined.

The aim of this study was to evaluate whether sarcopenia and myosteatosis are reliable predictors of clinical outcomes following emergency laparotomy in patients undergoing acute care surgery.

## 2. Materials and Methods

### 2.1. Study Design

We conducted a retrospective analysis of 324 patients who underwent EL at our institution, a tertiary referral teaching hospital in a metropolitan area, between January 2016 and December 2023.

**Figure 1:**
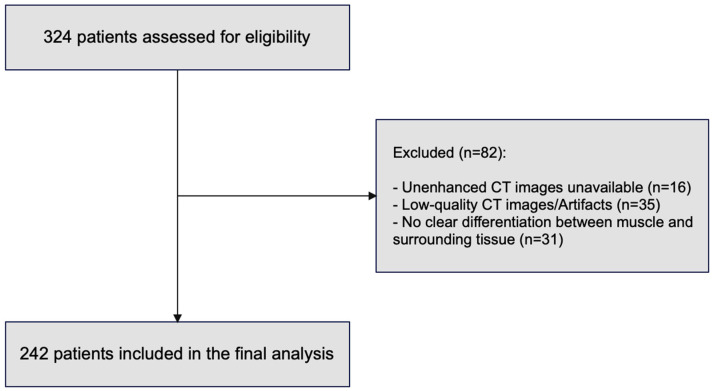
Study flowchart showing reasons for patient exclusion.

Inclusion criteria were as follows: age > 18 years and all emergency general surgery abdominal procedures performed via laparotomy. We excluded laparotomies performed for trauma and those in hemodynamically unstable patients, as abdominal CT was not performed in these cases. Eighty-two patients were excluded due to the unavailability of Skeletal Muscle Area (SMA), Skeletal Muscle Index (SMI), and Muscle Radiation Attenuation (MRA) measurements from Computed Tomography (CT) imaging. Two hundred and forty-two patients were included in the final analysis (Figure 1).

For each patient, age, sex, height (m), weight (Kg), body mass index (BMI), malignancy status, and preoperative laboratory values (White Blood Cells–WBC and C-Reactive Protein–CRP) have been recorded. Preoperative risk assessment included the American Society of Anesthesiologists (ASA) score [9], the Clinical Frailty Scale (CFS) [10], and the Charlson Comorbidity Index (CCI) [11]. The CCI was calculated using an online risk assessment tool, and patients were categorized into two groups: CCI ≥ 4 and CCI < 4. Similarly, patients were stratified according to CFS into three groups: A (CFS 1–3), B (CFS 4–5), and C (CFS ≥ 6).

Indications for laparotomy included bowel perforation, acute mesenteric ischemia, bowel obstruction, and other abdominal emergencies not falling into these major categories.

The primary outcomes were 30-day mortality and 30-day postoperative complications. The secondary outcomes were admission to the intensive care unit (ICU) and hospital length of stay (h-LOS). Postoperative complications were classified using the Clavien–Dindo (CD) classification [12], and only major complications (CD ≥ 3) were considered in the analysis.

### 2.2. Image Analysis

**Figure 2:**
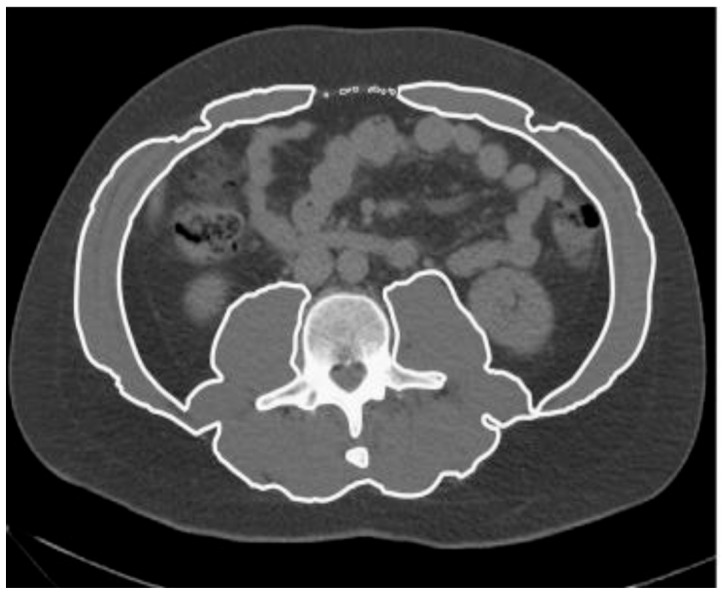
CT scan of a patient from our series showing the method for evaluating sarcopenia and myosteatosis: the slice considered corresponds to the level of the third lumbar vertebra; the skeletal muscle area is obtained from the combination of the psoas, paraspinal, and abdominal wall muscle areas.

All patients underwent a non-contrast-enhanced CT scan within 24–48 h prior to emergency laparotomy. A single axial image at the level of the third lumbar vertebra (L3) was selected for each patient. Skeletal muscle area (cm^2^) was quantified by manually outlining the psoas, paraspinal, and abdominal wall muscles, identified based on anatomical landmarks (Figure 2).

Total muscle area was normalized for stature (cm^2^/m^2^) to calculate the SMI. Sarcopenia was defined as an SMI ≤ 41.6 cm^2^/m^2^ for males and ≤ 32 cm^2^/m^2^ for females, based on established sex-specific cut-off values [13]. To define the sarcopenic status of patients, we considered only the radiological aspect, excluding the assessment of muscle strength, which could not be performed in an emergency setting.

Muscle radiodensity attenuation, expressed in Hounsfield Units (HU), was obtained by averaging the attenuation values of the selected muscle pixels. Myosteatosis was defined as an MRA ≤ 29.3 HU in males and ≤ 22 HU in females [13].

Image analysis was conducted using SYNAPSE Enterprise-PACS (Fujifilm Medical Systems, Tokyo, Japan).

### 2.3. Statistical Analysis

Data were collected using Microsoft Excel and analyzed with STATA software 18 (StataCorp, College Station, TX, USA).

Continuous variables were tested for normality using the Shapiro–Wilk test, summarized as Mean ± Standard Deviation or Medians (Interquartile Range–IQR) and compared using Student’s *t*-test or the Mann–Whitney U test, as appropriate. Categorical variables were reported as frequencies and percentages and compared using the χ^2^ test or Fisher’s exact test.

Univariable regression models were performed for each outcome. Binary outcomes (30-day mortality, severe postoperative complications, and ICU admission) were analyzed with logistic regression, reporting odds ratios (OR) with 95% confidence intervals (CI). H-LOS was analyzed as a time-to-event outcome (time to discharge) using Cox proportional-hazards regression, reporting hazard ratios (HR) with 95% CI with HR > 1 for faster discharge, i.e., shorter LOS. Variables with a *p*-value < 0.05 in univariate analysis were included in the multivariate regression model to adjust for confounding factors.

Variables with *p* < 0.05 in univariable analysis were further included in multivariable models.

Collinearity between evaluated clinical scores (ASA, CCI, CFS) was assessed using the variance inflation factor (VIF) and corresponding tolerance values (1/VIF).

The surgical diagnosis was modeled as a categorical variable; effect estimates were reported for each category, and a global ***p***-value was obtained using Wald tests.

A two-tailed *p*-value < 0.05 was considered statistically significant for all the performed statistical analyses, except for the univariate one, as previously stated.

## 3. Results

### 3.1. Patient Characteristics

A total of 242 patients underwent EL. The cohort included 124 males and 118 females aged between 21 and 96 years (median age of 70 years), with a median BMI of 23.9 kg/m^2^. Active cancer was present in 36.8%, and 63.2% had a CCI ≥ 4. Most were ASA grade 3 (50.4%), and 52.9% were classified as CFS category A. The most common indication for EL in both groups was intestinal obstruction, occurring in 43.8%. A detailed summary of patient demographics is provided in Table 1.

### 3.2. Sarcopenic and Myosteatosic Patient Subgroups

The median values of SMA, SMI, and MRA were 107.1 cm^2^, 39.1 cm^2^/m^2^, and 14.75 HU, respectively (Table 2). Based on body composition analysis, 103 patients (42.6%) were considered sarcopenic and 189 (78.1%) myosteatosic. Patient characteristics by sarcopenia and myosteatosis status are detailed in Table 3.

### 3.3. Mortality

Thirty-day mortality was 23.1% (Table 4). In univariate analysis, CCI ≥ 4 (*p* = 0.018) and higher CFS (*p* < 0.001) were significantly associated with postoperative in-hospital mortality, while sarcopenia and myosteatosis were not (Table 5). In multivariable analysis, mesenteric ischemia (OR 2.77, 95% CI 1.11–6.87, *p* = 0.035) and higher CFS (OR 2.46, 95% CI 1.15–5.24, *p* = 0.021) remained independent predictors of mortality.

### 3.4. Severe Complications

Severe postoperative complications occurred in approximately 34% of patients (Table 4). These were significantly associated with myosteatosis (*p* = 0.048), higher ASA score (*p* = 0.013), CRP (*p* = 0.002), and higher CFS (*p* = 0.002). Patients undergoing EL for bowel obstruction had a lower rate of severe complications (*p* < 0.001), confirmed also as an independent predictor at multivariable analysis (OR 0.31, 95% CI: 0.15–0.64, *p* = 0.011), as shown in Table 5.

### 3.5. ICU Admission

ICU admission was required in 43.8% of cases (Table 4). At univariate analysis, associations were significant for age (*p* = 0.015), ASA (*p* < 0.001), CCI ≥ 4 (*p* = 0.001), CFS (*p* < 0.001), and myosteatosis (*p* = 0.009). Mesenteric ischemia had the highest ICU admission rate (*p* < 0.001) among the procedures. Multivariate analysis (Table 5) confirmed the role of ASA score (OR 1.17, 95% CI: 1.1–2.64, *p* = 0.016) and mesenteric ischemia (OR 4.96, 95% CI: 1.47–16.75, *p* = 0.01) as independent predictors, while bowel obstruction was associated with reduced ICU admission (OR 0.32, 95% CI: 0.15–0.66, *p* < 0.002).

### 3.6. Length of Hospitalization

The median h-LOS was 15 days (Table 4). At univariable analysis, factors significantly associated with prolonged h-LOS included myosteatosis (*p* = 0.007), ASA score (*p* = 0.015), and CFS (*p* = 0.029). Myosteatosis was identified as an independent predictor of longer h-LOS in the multivariable model (HR 0.59, 95% CI: 0.42–0.84, *p* = 0.003). Conversely, mesenteric ischemia (HR 1.83, 95% CI: 1.05–3.19, *p* = 0.032) and bowel obstruction (HR 1.99, 95% CI: 1.51–3.17, *p* = 0.004) were associated with a shorter h-LOS, as shown in Table 5.

## 4. Discussions

The wide heterogeneity of patients, underlying diseases, and indications, as well as the urgency of the situation itself, make predicting outcomes in emergency surgery particularly challenging. In fact, emergency laparotomy is intrinsically associated with a high risk of morbidity, disability, and mortality [14]. Jansson et al. [15] reported an overall short-term mortality rate of 14.2%, which is comparable to other recent studies in the field (ranging from 7% to 21%) [16] and to our series, in which the 30-day mortality rate was 23.1%.

Several scoring systems have been proposed to evaluate outcomes following EL; however, they can vary considerably in accuracy and applicability [2,3,17,18].

The evaluation of SMI and MRA appears to enable a more accurate assessment of the risk of mortality and treatment-related complications, potentially allowing for the identification of patients at increased risk and the implementation of necessary preoperative strategies [19,20,21].

The prevalence of sarcopenia and myosteatosis observed in our study was higher than that reported in other series available in the literature [22,23]. This finding can likely be attributed to the relatively low cut-off values we selected for our analysis, as well as to the slightly higher median age of our study population. Consistent with previous studies, we also confirmed that older patients with more comorbidities and active oncologic disease are more frequently affected by sarcopenia [24,25]. These individuals are particularly vulnerable and, especially in the emergency setting, require special attention. Similarly, patients with myosteatosis tend to be older and have multiple comorbidities. Moreover, our data suggest a strong correlation between myosteatosis and overall levels of fitness and frailty, supporting the hypothesis that changes in fat distribution are associated with impairments in autonomy and physical function [26].

In our analysis, preoperative SMI and MRA did not demonstrate a significant association with 30-day mortality after emergent open abdominal surgery, as also reported in the study by Thu et al. [27]. These findings differ from previous studies: Body et al. [26] identified sarcopenia and myosteatosis as independent predictors of 30-day mortality, Wu et al. [28] confirmed similar results, and Francomacaro et al. [29] reported an association between sarcopenia alone and increased postoperative mortality and severe complications. Our data, however, suggest that these associations may not be universally applicable, particularly in the emergency setting, where multiple confounding factors can mitigate the prognostic impact of sarcopenia and myosteatosis.

Clinical Frailty Scale proved to be an accurate and reliable predictor of short-term mortality in patients undergoing emergency surgery, consistent with previous findings [30]. Similarly, Parmar et al. demonstrated that frailty, also measured with the CFS, was associated with increased 30- and 90-day mortality in a comparable population undergoing emergency laparotomy [31]. Other previous studies further support the role of preoperative frailty assessment as a valuable tool to predict adverse postoperative outcomes [32,33].

Our findings indicate that myosteatosis, but not sarcopenia alone, is associated with prolonged hospital stay after emergency laparotomy. This observation is in line with previous evidence, as Salem et al. identified MRA as an independent predictor of extended hospital length of stay in surgical patients [20], a result confirmed by Evans et al. [34]. The underlying mechanism may involve poor nutritional status and an increased vulnerability to postoperative complications, both of which are recognized contributors to delayed recovery and prolonged hospitalization.

Therefore, identifying vulnerable patients preoperatively allows surgeons to tailor counseling, consider less invasive strategies, and implement dedicated care pathways, including specialized anesthesia protocols, critical care management, and perioperative nutritional or exercise rehabilitation interventions.

In univariate analysis, patients with higher ASA scores and higher CFS values were those most vulnerable to postoperative complications. Few studies [8,35] have specifically assessed the impact of myosteatosis on complications following cancer surgery. In our cohort, myosteatosis was associated with a higher incidence of overall and severe complications. However, in the multivariate analysis, MRA did not emerge as an independent predictor of severe complications. Additionally, patients who underwent EL for intestinal obstruction experienced significantly fewer complications compared to those who had surgery for other indications. Therefore, when evaluating outcomes after emergency laparotomy, it is crucial to take into account not only the patient’s overall clinical status but also the specific indication leading to the urgent surgical intervention.

In the univariate analysis, patients with myosteatosis were more likely to require ICU admission after emergency surgery; however, this association was not confirmed in the multivariate model. Conversely, and in contrast to the meta-analysis by Tao-ran Yang et al. [23], our series did not demonstrate a correlation between sarcopenia and ICU admission. The ASA score emerged as an independent predictor of ICU admission, as did intestinal ischemia as an indication for emergency laparotomy. In the latter condition, intensive care admission is often an integral component of the treatment strategy, which typically includes open abdomen management and a planned “second-look” procedure following the initial surgery. Moreover, these patients frequently present with severe physiological derangements requiring stabilization and continuous intensive monitoring [36]. While patients with bowel ischemia commonly need intensive postoperative care, those with intestinal obstruction rarely do.

Taken together, our findings suggest that patients with myosteatosis and those undergoing emergency laparotomy for intestinal ischemia are at increased risk of ICU admission, which translates into higher healthcare costs and a greater clinical burden.

### Limitations of the Study

Several limitations should be acknowledged when interpreting the findings of this study. First, due to its retrospective design, data were extracted from patients’ medical records, which may have affected the accuracy of the analyses and introduced potential bias in the evaluation of functional outcomes.

Second, the sample size was relatively small and exhibited considerable heterogeneity in terms of patient characteristics, surgical procedures, and operative indications. A prospective study, in which standardized data collection can be ensured, would be necessary to validate these findings.

Additionally, the majority of patients in our cohort were classified as sarcopenic and/or myosteatotic. In this study, we adopted threshold values for SMA, SMI, and MRA based on body composition data from a relatively small cohort of healthy Caucasian individuals [4]. However, standardized cut-off values are not yet established in the literature, and no consensus currently exists. As such, some patients identified as sarcopenic or myosteatotic in our analysis might have been classified differently using alternative reference values. This highlights the need for further validation of these criteria in larger, prospective cohorts.

Finally, CT image analysis was conducted by multiple radiologists. While these measurements are considered objective and reproducible, inter-observer variability may occur in the delineation of target areas. To enhance measurement precision and consistency, future studies would benefit from evaluations performed by a single, experienced radiologist.

## 5. Conclusions

Our findings indicate that sarcopenia and myosteatosis do not significantly influence 30-day mortality in patients undergoing emergency laparotomy. Existing scoring systems may provide better prediction of short-term mortality in this setting.

Importantly, myosteatosis appears to be a valid marker of frailty and may help identify patients at higher risk of prolonged hospitalization.

While recognizing high-risk patients does not alter the need for urgent surgical intervention, this information could guide perioperative management, enabling tailored preoperative counseling, consideration of less invasive approaches, and implementation of specialized care pathways.

## Figures and Tables

**Table 1:** Characteristics of the patients.

Patient Characteristics	Whole Cohort (*n* = 242)
	***n* (%)**/Median [IQR]
Age, (years)	70 (58–78)
Gender	
F	118 (48.8)
M	124 (51.2)
BMI, (kg/m^2^)	23.9 [21.1–26.9]
Oncologic disease	
Yes	89 (36,8)
No	153 (63,2)
White blood cell count, (×10^3^/μL)	11.1 [7.7–16.2]
ASA Physical Status	
1	26 (10.7)
2	59 (24.4)
3	122 (50.4)
4	35 (14.5)
Charlson Comorbidity Index	
<4	89 (36.8)
≥4	153 (63.2)
Clinical Frailty Scale	
A (1–3)	128 (52.9)
B (4–5)	74 (30.6)
C (≥6)	40 (16.5)
Indication for surgery	
Bowel perforation	82 (33.9)
Mesenteric Ischemia	27 (11.1)
Bowel Occlusion	106 (43.8)
Other	27 (11.2)

IQR, Interquartile Range. BMI: Body Mass Index; ASA: American Association of Anesthesiology.

**Table 2:** Sex-specific threshold values for selected body composition measurements at the third lumbar vertebra.

	Male (124)		Female (118)		Overall (242)
	Median (IQR)	Threshold Value	Median (IQR)	Threshold Value	Median (IQR)
Skeletal muscle area–SMA (cm^2^)	112.8 [90.2–129.1]	134	102.9 [79.9–127.4]	89,2	107.1 [84.6–128.6]
Skeletal muscle index–SMI (cm^2^/m^−2^)	41,1 [36.7–46.3]	41.6	35.5 [6.71–175.5]	32	39,1 [32.5–44.9]
Muscle radiation attenuation–MRA (HU)	19 [6.0–27.5]	29.3	9.5 [5.0–19.0]	22	14.8 [0.0–25.2]

HU, Hounsfield Unit; IQR, Interquartile Range.

**Table 3:** Characteristics of patients with and without sarcopenia and myosteatosis.

Patient Characteristics	Sarcopenia			Myosteatosis		
	*n* (%)/Median [IQR]		*p* Value	*n* (%)/Median [IQR]		*p* Value
	Yes	No		Yes	No	
	(*n* = 103, 42.6%)	(*n* = 139, 57.4%)		(*n* = 189, 78.1%)	(*n* = 53, 21.9%)	
Age (years)	71 [60–80]	69 [57–77]	0.067	72 [65–80]	52 [41–63]	**<0.001**
Gender			**0.001**			0.793
F	38 (36.9)	80 (57.6)		93 (49.2)	25 (47.2)	
M	65 (63.1)	59 (42.5)		96 (50.8)	28 (52.8)	
BMI (kg/m^2^)	21.7	25.7	**<0.001**	24.8	21.7	**<0.001**
	[19.4–24.8]	[23.0–28.7]		[21.7–27.3]	[19.5–24.2]	
Oncologic disease			**0.006**			0.627
Yes	48 (46.6)	41 (29.5)		68 (36.0)	21 (39.6)	
No	55 (53.4)	98 (70.5)		121 (64.0)	32 (60.4)	
Lab data						
WBC (×10^3^/μL)	11.14	11.1	0.334	11.4	10.8	0.751
	[7.0–16.1]	[8.0–16.6]		[7.5–16.6]	[8.1–14.8]	
CRP (mg/dL)	8.7	9.5	0.543	11.1	1.76 [0.61–9.5]	**0.001**
	[1.4–21.0]	[2.1–20.7]		[2.5–21.8]		
ASA Score			0.192			**<0.001**
1	6 (5.8)	20 (14.4)		15 (7.9)	11 (20.8)	
2	25 (24.3)	34 (24.5)		38 (20.1)	21 (39.6)	
3	56 (54.4)	66 (47.5)		105 (55.6)	17 (32.1)	
4	16 (15.5)	19 (13.7)		31 (16.4)	4 (7.6)	
CCI			**0.001**			**<0.001**
<4	26 (25.2)	63 (45.3)		55 (29.1)	34 (64.2)	
≥4	77 (74.8)	76 (54.7)		134 (70.9)	19 (35.9)	
CFS			0.301			**0.001**
A (1–3)	50 (48.5)	79 (56.8)		89 (47.1)	40 (75.5)	
B (4–5)	33 (32.0)	42 (30.2)		65 (34.4)	10 (18.9)	
C (≥6)	20 (19.4)	18 (13.0)		35 (18.5)	3 (5.7)	

BMI, Body Mass Index; ASA, American Society of Anesthesiologists; CCI, Charlson Comorbidity Index; CFS, Clinical Frailty Scale; WBC, White Blood Cell count, ×10^3^/μL; CRP, C-Reactive Protein.

**Table 4:** Comparison of clinical outcomes between groups according to sarcopenia and myosteatosis.

Outcome	Overall	Sarcopenia			Myosteatosis		
	*n* (%)/Median [IQR]	*n* (%)/Median [IQR]		*p* Value	*n* (%)/Median [IQR]		*p* Value
	(*n* = 242)	Yes	No		Yes	No	
		(*n* = 103, 42.6%)	(*n* = 139, 57.4%)		(*n* = 189, 78.1%)	(*n* = 53, 21.9%)	
30-day Mortality	56 (23.1)	27 (26.1)	29 (20.9)	0.329	48 (25.4)	8 (15.1)	0.116
Length of hospital stay	15 [8–30]	16 [9–30]	14 [7–31]	0.554	17 [9–34]	8 [6–16]	**<0.001**
ICU admission	106 (43.8)	46 (44.7)	60 (43.1)	0.817	91 (48.2)	15 (28.3)	**0.010**
Severe Complications	82 (33.9)	41 (39.9)	41 (29.5)	0.094	70 (37.1)	12 (22.7)	**0.048**

ICU, Intensive Care Unit.

**Table 5:** Univariate and multivariate analysis of factors associated with mortality, length of hospitalization, severe complications, and ICU admission.

**Mortality**	**Univariate OR**	**95% CI**	***p* Value**	**Multivariate OR**	**95% CI**	***p* Value**
Age (years)	1.02	[0.96–1.04]	0.078	e	e	e
Gender (male vs. female)	0.57	[0.32–1.04]	0.063	e	e	e
BMI (kg/m^2^)	1.01	[0.96–1.06]	0.723	e	e	e
Oncologic status	1.04	[0.56–1.93]	0.131	e	e	e
CRP	0.99	[0.97–1.02]	1.024	e	e	e
WBC	1.01	[0.97–1.04]	0.677	e	e	e
CCI	2.28	[1.15–4.54]	**0.018**	1.62	[0.61–4.3]	0.321
CFS	3.02	[1.59–5.72]	**<0.001**	2.46	[1.15–5.24]	**0.021**
ASA score	1.38	[0.95–2.00]	0.087	0.95	[0.6–1.49]	0.874
Bowel perforation	0.49	[0.18–1.34]	0.438	e	e	e
Mesenteric Ischemia	3.1	[1.35–7.11]	**0.007**	2.77	[1.11–6.87]	**0.035**
Bowel occlusion	0.62	[0.24–1.61]	0.327	e	e	e
Other Diagnosis	1.65	[0.56–1.46]	0.338	e	e	e
Sarcopenia	1.35	[0.74–2.46]	0.336	e	e	e
Myosteatosis	1.91	[0.84–4.34]	0.122	e	e	e
**Severe complications**	**Univariate OR**	**95% CI**	***p* value**	**Multivariate OR**	**95% CI**	***p* value**
Age (years)	1.01	[0.99–1.09]	0.450	e	e	e
Gender (male vs. female)	0.86	[0.51–1.47]	0.509	e	e	e
BMI (kg/m^2^)	1.00	[0.97–1.05]	0.903	e	e	e
Oncologic status	1.35	[0.78–2.34]	0.283	e	e	e
CRP	1.03	[1.01–1.05]	**0.002**	1.01	[0.98–1.03]	0.414
WBC	1.02	[0.99–1.06]	0.102	e	e	e
CCI	1.04	[0.97–1.12]	0.297	e	e	e
CFS	1.27	[1.09–1.48]	**0.002**	1.14	[0.93–1.41]	0.205
ASA score	1.52	[1.09–2.13]	**0.013**	1.3	[0.91–1.85]	0.143
Bowel Perforation	0.57	[0.24–1.36]	0.204	e	e	e
Mesenteric Ischemia	2.76	[1.22–6.22]	**0.013**	1.5	[0.62–3.6]	0.362
Bowel Occlusion	0.23	[0.12–0.42]	**<0.001**	0.31	[0.15–0.64]	**0.011**
Other Diagnosis	1.58	[0.92–2.70]	0.081	e	e	e
Sarcopenia	1.58	[0.92–2.70]	0.095	e	e	e
Myosteatosis	2.0	[1.12–4.07]	**0.048**	1.3	[0.61–2.88]	0.467
**ICU admission**	**Univariate OR**	**95% CI**	***p* value**	**Multivariate OR**	**95% CI**	***p* value**
Age (years)	1.02	[1.01–1.04]	**0.015**	1.01	[0.98–1.04]	0.472
Gender (male vs. female)	1.24	[0.75–2.08]	0.39	e	e	e
BMI (kg/m^2^)	1.02	[0.97–1.06]	0.48	e	e	e
Oncologic status	1.24	[0.73–2.10]	0.418	e	e	e
CRP	1.03	[1.01–1.05]	**0.002**	1.01	[0.99–1.04]	0.312
WBC	1.02	[0.99–1.06]	0.167	e	e	e
CCI	2.45	[1.41–4.26]	**0.001**	1.31	[0.6–2.89]	0.517
CFS	3.66	[1.97–6.76]	**<0.001**	1.71	[0.8–3.67]	0.171
ASA score	2.33	[1.63–3.32]	**<0.001**	1.17	[1.1–2.64]	**0.016**
Bowel Perforation	0.62	[0.25–1.51]	0.145	e	e	e
Mesenteric Ischemia	9.1	[3.05–27.4]	**<0.001**	4.96	[1.47–16.75]	**0.013**
Bowel Occlusion	0.19	[0.1–0.34]	**<0.001**	0.32	[0.15–0.66]	**<0.002**
Other Diagnosis	1.33	[0.84–1.45]	0.081	e	e	e
Sarcopenia	1.06	[0.64–1.78]	0.816	e	e	e
Myosteatosis	2.35	[1.21–4.56]	**0.010**	0.89	[0.36–2.21]	0.821
**Length of hospitalization**	**Univariate HR**	**95% CI**	***p* value**	**Multivariate HR**	**95% CI**	***p* value**
Age (years)	1.00	[0.99–1.01]	0.982	e	e	e
Gender (male vs. female)	0.96	[0.74–1.23]	0.736	e	e	e
BMI (kg/m^2^)	0.99	[0.97–1.01]	0.283	e	e	e
Oncologic status	0.95	[0.73–1.11]	0.723	e	e	e
CRP	0.98	[0.97–0.99]	**0.001**	0.99	[0.99–1.01]	0.352
WBC	0.98	[0.97–1.00]	**0.018**	0.99	[0.97–1.00]	0.106
CCI	0.98	[0.96–1.03]	0.585	e	e	e
CFS	0.91	[0.84–0.99]	**0.029**	1.02	[0.92–1.14]	0.683
ASA score	0.80	[0.69–0.94]	**0.015**	0.83	[0.69–1.00]	0.052
Bowel Perforation	1.29	[0.83–2.00]	0.261	e	e	e
Mesenteric Ischemia	1.76	[1.03–3.05]	**0.038**	1.83	[1.05–3.19]	**0.032**
Bowel Occlusion	2.32	[1.51–3.57]	**<0.001**	1.99	[1.24–3.17]	**0.004**
Other Diagnosis	1.08	[0.84–1.4]	0.548	e	e	e
Sarcopenia	1.08	[0.84–1.40]	0.398	e	e	e
Myosteatosis	0.65	[0.48–0.89]	**0.007**	0.59	[0.42–0.84]	**0.003**

ASA: American Society of Anesthesiologists, BMI: Body Mass Index, CCI: Charlson Comorbidity Index, CI: Confidence Interval, CRP: C-Reactive Protein, CFS: Clinical Frailty Scale, HR: Hazard Ratio, ICU: Intensive Care Unit, OR: Odds Ratio, WBC: White Blood Cells, e: Excluded From The Multivariate Analysis.

## Data Availability

The raw data supporting the conclusions of this article will be made available by the authors on request.

## Abbreviations

The following abbreviations are used in this manuscript:

EL	Emergency Laparotomy
SMA	Skeletal Muscle Area
SMI	Skeletal Muscle Index
MRA	Muscle Radiation Attenuation
CT	Computed Tomography
BMI	Body Mass Index
ASA	American Society of Anesthesiologists
CFS	Clinical Frailty Scale
CCI	Charlson Comorbidity Index
ICU	Intensive Care Unit
h-LOS	hospital Length Of Stay
CD	Clavien-Dindo
HU	Hounsfield Units
